# The Research Defence Society

**Published:** 1913-05-17

**Authors:** David Gill, Sydney Holland, Arthur R. Cushny, Eldred Horsley, Reginald Talbot, Beatrice Leslie Thomson, F. M. Sandwith, Stephen Paget

**Affiliations:** President.; Vice-President.; Chairman of Committee.; Member of Committee.; Member of Committee.; Member of Committee.; Member of Committee.; Hon. Treasurer.; Hon. Secretary.


*To the Editor of* THE HOSPITAL.

### The Research Defence Society.

*To the Editor of* THE HOSPITAL.

SIR,—We have carefully considered what course our Society ought to pursue in view of the recent exposure of anti-vivisection. We are of opinion that the Society ought to maintain, for some years, in London, a place of its own, where it could put before the public, every day and all day long, the truth about experiments on animals in this country. We have tried this method in temporary premises, and have found it abundantly successful, and we now have the opportunity of acquiring a small ground floor and basement, at a moderate rent, in one of the more important thoroughfares, admirably suited for our work. It is the plain duty of all who desire the prevention of disease among mankind and among animals to help "the man in the street" to arrive at a right judgment in this matter. We are convinced from experience that the best way of dealing with anti-vivisection methods at the present time is to maintain a bureau where all passers-by can read and learn for themselves the truth about experiments on animals, and can obtain pamphlets and leaflets, and ask for further information.

We therefore appeal for generous contributions toward a special fund for this one purpose. It is the especial business of the Research Defence Society to dispel the ignorance, or worse than ignorance, which still exists as to the character and the object of experiments on animals in this country; and the maintenance of a bureau in London would be of an immense advantage to the work of the Society. All donations and subscriptions should be sent to the Hon. Treasurer, 21 Ladbroke Square, London, W. All cheques should be crossed Messrs. Coutts and Co.—We remain, yours faithfully,

DAVID GILL, President.
CROMER, Vice-President.
SYDNEY HOLLAND, Chairman of Committee.
ARTHUR R. CUSHNY, Member of Committee.
ELDRED HORSLEY, Member of Committee.
REGINALD TALBOT, Member of Committee.
BEATRICE LESLIE THOMSON, Member of Committee.
F. M. SANDWITH, Hon. Treasurer.
STEPHEN PAGET, Hon. Secretary.

May 5, 1913.

